# Analysis of Gene Expression Using Gene Sets Discriminates Cancer Patients with and without Late Radiation Toxicity

**DOI:** 10.1371/journal.pmed.0030422

**Published:** 2006-10-31

**Authors:** J. Peter Svensson, Lukas J. A Stalpers, Rebecca E. E. Esveldt–van Lange, Nicolaas A. P Franken, Jaap Haveman, Binie Klein, Ingela Turesson, Harry Vrieling, Micheline Giphart-Gassler

**Affiliations:** 1 Department of Toxicogenetics, Leiden University Medical Center, Leiden, Netherlands; 2 Department of Oncology, Radiology, and Clinical Immunology, Academic Hospital, Uppsala, Sweden; 3 Department of Radiotherapy/LEXOR Laboratory of Experimental Oncology and Radiobiology, Academic Medical Center, University of Amsterdam, Amsterdam, Netherlands; Netherlands Cancer Institute, Netherlands

## Abstract

**Background:**

Radiation is an effective anti-cancer therapy but leads to severe late radiation toxicity in 5%–10% of patients. Assuming that genetic susceptibility impacts this risk, we hypothesized that the cellular response of normal tissue to X-rays could discriminate patients with and without late radiation toxicity.

**Methods and Findings:**

Prostate carcinoma patients without evidence of cancer 2 y after curative radiotherapy were recruited in the study. Blood samples of 21 patients with severe late complications from radiation and 17 patients without symptoms were collected. Stimulated peripheral lymphocytes were mock-irradiated or irradiated with 2-Gy X-rays. The 24-h radiation response was analyzed by gene expression profiling and used for classification. Classification was performed either on the expression of separate genes or, to augment the classification power, on gene sets consisting of genes grouped together based on function or cellular colocalization.

X-ray irradiation altered the expression of radio-responsive genes in both groups. This response was variable across individuals, and the expression of the most significant radio-responsive genes was unlinked to radiation toxicity. The classifier based on the radiation response of separate genes correctly classified 63% of the patients. The classifier based on affected gene sets improved correct classification to 86%, although on the individual level only 21/38 (55%) patients were classified with high certainty. The majority of the discriminative genes and gene sets belonged to the ubiquitin, apoptosis, and stress signaling networks. The apoptotic response appeared more pronounced in patients that did not develop toxicity. In an independent set of 12 patients, the toxicity status of eight was predicted correctly by the gene set classifier.

**Conclusions:**

Gene expression profiling succeeded to some extent in discriminating groups of patients with and without severe late radiotherapy toxicity. Moreover, the discriminative power was enhanced by assessment of functionally or structurally related gene sets. While prediction of individual response requires improvement, this study is a step forward in predicting susceptibility to late radiation toxicity.

## Abstract 2

Expression profiling can discriminate between groups of patients with and without severe late radiotherapy toxicity but not (yet) predict individual responses.

## Editors' Summary

**Background.:**

More than half the people who develop cancer receive radiotherapy as part of their treatment. That is, tumor cells are destroyed by exposing them to a source of ionizing radiation such as X-rays. Ionizing radiation damages the genetic material of cancer cells so that they can no longer divide. Unfortunately, it also damages nearby normal cells, although they are less sensitive to radiation than the cancer cells. Radiotherapists minimize how much radiation hits normal tissues by carefully aiming the X-rays at the tumor. Even so, patients often develop side effects such as sore skin or digestive problems during or soon after radiotherapy; the exact nature of the side effects depends on the part of the body exposed to the X-rays. In addition, a few patients develop severe late radiation toxicity, months or years after their treatment. Like early toxicity, late toxicity occurs in the normal tissues near the tumor site. For example, in prostate cancer—a tumor that forms in a gland in the male reproductive system that lies between the bladder and the end of the gut (the rectum)—late radiation toxicity affects rectal, bladder, and sexual function in 5%–10% of patients.

**Why Was This Study Done?:**

It is not known why some patients develop late radiation toxicity, and it is impossible to predict before treatment which patients will have long-term health problems after radiotherapy. It would be useful to know this, because radiation levels might be reduced in those patients, while larger doses of radiation could be given to patients at low risk of late complications to ensure a complete eradication of their cancer. One theory is that some patients are genetically predisposed to develop severe late radiation toxicity. In other words, their genetic make-up makes it more likely that their tissues develop long-term complications after radiation damage. In this study, the researchers looked for markers of a genetic predisposition for late radiation toxicity by comparing radiation-induced changes in the pattern of cellular proteins in patients who had late radiation toxicity after radiotherapy with the changes seen in patients who did not develop such complications.

**What Did the Researchers Do and Find?:**

The researchers recruited 38 patients who had been treated successfully with radiotherapy for prostate cancer two years previously. Of these, 21 had developed severe late radiation toxicity. They isolated lymphocytes (a type of immune system cell) from the patients' blood, stimulated the lymphocytes to divide, exposed them to X-rays, and analyzed the pattern of genes active in these cells—their gene expression profile—before and after irradiation. The researchers found that irradiation induced the expression of numerous genes in the lymphocytes, including many well-known radiation-responsive genes. They then used an analytical process called “random cross-validation” to look for a gene expression profile (or molecular signature) that was associated with late radiation toxicity. They report that a signature based on the radiation response of 50 individual genes correctly classified 63% of the patient population in terms of whether the patient had developed late radiation toxicity. A signature based on the radiation response of gene sets containing genes linked by function or cellular localization correctly classified 86% of the patient population.

**What Do These Findings Mean?:**

Gene expression profiling identified groups of patients who had had severe late radiation toxicity pretty well, particularly when sets of related genes were used to classify the patients. The approach was not so good, however, at identifying individual patients who had had problems, being correct and certain only half the time. Additional studies are needed, therefore, before this promising approach can be used clinically to predict patient responses to radiotherapy. Overall, the study supports the idea that some patients are genetically predisposed to develop late radiation toxicity, and it also provides clues about which cellular pathways help to determine late radiation toxicity. Most of the genes and gene sets that discriminated between the patients with and without late radiation toxicity are involved in protein metabolism, apoptosis (a special sort of cell death), and stress signaling networks (pathways that protect cells from damage). This information, if confirmed, might help researchers to develop therapeutic interventions to minimize late radiation toxicity in vulnerable individuals.

**Additional Information.:**

Please access these Web sites via the online version of this summary at http://dx.doi.org/10.1371/journal.pmed.0030422.

- US National Cancer Institute patient information on radiotherapy and on prostate cancer
- American Cancer Society information on radiation therapy
- Cancer Research UK patient information on radiotherapy
- Wikipedia pages on radiotherapy (note that Wikipedia is a free online encyclopedia that anyone can edit)

## Introduction

Radiotherapy is one of the most effective treatments for cancer. The incidence of prostate cancer is high, but most patients will be cured or free from tumor symptoms within several years after radiotherapy. The success of radiotherapy depends on its ability to kill cancer cells while sparing normal tissue [1]. Toxicity risk is affected by radiation dose and volume as well as age and condition of the patient. Clinical trials have demonstrated that escalation of the radiation dose increases local tumor control [2–4] but may also increase the risk of late complications [5]. Late toxicity after radiotherapy for prostate cancer includes disturbed rectal, bladder, and sexual functions. The percentage of patients developing severe late toxicity determines the maximum acceptable radiation dose. Generally an adverse effect frequency of 5%–10% is considered acceptable. Many more patients develop minor toxicity, and only a few remain symptom-free.

Radiation risk can only be partly explained by clinical factors such as age, condition of the patient, and radiation dose and volume. There is evidence of large patient-to-patient variability in the development of late complications [6–8], suggesting the existence of additional risk factors. There is increasing evidence that genetic predisposition is a determining factor [7]. In the past decade, several research groups have tried to develop assays for predicting radiation toxicity in normal tissues [8–14]. However, the resulting data are contradictory, and correlations observed in most studies are at best marginally significant [15]. Frequencies of chromosomal aberrations in ex vivo irradiated peripheral blood lymphocytes are generally increased in patients displaying normal tissue toxicity after radiotherapy. However, the correlation is too weak to allow pretreatment identification of such patients [16].

The individual variability in normal tissue response after radiotherapy may be caused by subtle mutations in genes involved in the cellular response to radiation. Genotoxic stress has been shown to induce massive alterations at the transcriptional level [17]. Investigation of the transcriptional response by gene expression profiling may therefore be a suitable approach to identify individuals with a genetic predisposition for late radiation toxicity. Recently, it has been shown that variation in the expression level of many genes has a heritable component [18].

Rieger and co-workers used gene expression profiling with microarrays to characterize and classify a diverse group of cancer patients with acute radiation toxicity [19]. They concluded that genes involved in the response to DNA damage, including apoptosis and ubiquitin genes, were associated with the observed clinical toxicity. However, the genes associated with radiation toxicity were not the most pronounced radiation responsive genes described in several other studies [20–22]. There are additional promising gene expression profiling studies in oncology [23,24], but the applicability of their findings is—to an extent—limited [25]. Because of the sheer number of genes being monitored, it is often possible to find highly specific settings that provide an optimal and acceptable misclassification rate. To avoid some of this bias, Michiels and co-workers proposed a random validation strategy for classification [25]. However, some commentators have suggested, based on this work, that thousands of patients would be required to thoroughly validate a microarray classifier [26,27].

An alternative approach is a classification strategy based on functional modules made up of multiple genes [28–31]. This approach might better accommodate the existing genetic heterogeneity within a patient group. The joint behavior of functionally or spatially related genes may be significant, whereas the activity of individual genes may not. Another advantage of this approach is that it might lead to a more relevant biological interpretation of the results.

In our study we used gene expression profiling to try to discriminate prostate cancer patients with severe late radiation complications following radiotherapy (over-responders [ORs]) from patients without such complications (non-responders [NRs]). The study was restricted to the extreme responders to optimize the chances of success. For late normal tissue toxicity, prostate cancer patients are advantageous to study as they form a relatively homogeneous group. Confounding factors are limited since there is no gender effect, age variability is relatively small, and the only cancer treatment adjunctive to radiotherapy is medical or surgical castration.

## Methods

This study was approved by the local medical ethical committee.

### Patient Selection

Late toxicity was recorded in 800 patients with prostate cancer irradiated at the Academic Medical Center of University of Amsterdam, the Netherlands, between 1996 and 2003 using the EORTC SOMA scale [32]. Patients with no clinical progression or no prostate-specific antigen rise 2 y after curative external beam radiotherapy were selected. Patients with T1 and T2 tumors received 70 Gy of conformal external beam prostate radiotherapy; patients with T3 and T4 tumors received elective pelvic radiotherapy (40 or 50 Gy) plus a conformal boost of the prostate to 70 Gy, and 3 y of androgen deprivation therapy. Patients were preselected based on recorded toxicity grading, which was reconfirmed during a standard follow-up appointment. ORs were defined as patients with grade III toxicity to the bladder and/or rectum. Different from the SOMA scale, patients with severe or frequent blood loss requiring medical intervention were also classified as OR with grade III toxicity. NRs were defined as patients that experienced no adverse effects (grade 0 toxicity). Some of the patients previously recorded “without” toxicity had minor (grade I) or moderate (grade II) toxicity on careful re-interviewing. Based on the data registration, corrected by out-patient reconfirmation, the estimated prevalence of grade 0 toxicity was 5%–10%, of grade I or II toxicity was 80%–90%, and of grade III toxicity was 5%–10%. Life-threatening toxicity was not reported.

Following written informed consent, blood samples were collected from 50 donors. Thirty-eight patients—21 ORs and 17 NRs—participated in the primary classification study. Twelve patients (six ORs and six NRs) were used for an independent validation. From the ORs, 21 had severe proctitis, two had severe proctitis and cystitis, one had cystitis only, one had small bowel ileus, and two had rectal fibrosis.

### Sample Preparation

Lymphocytes were isolated from whole blood using Ficoll (Ficoll-Paque PLUS, Amersham Biosciences [now GE Healthcare], http://www.amershambiosciences.com) gradient separation. Freshly isolated T cells at a cell density of 5 × 10^5^ cells/ml were stimulated for 44 h [33] with phytohemagglutinin at a final concentration of 1 μg/ml. In the validation set, only frozen lymphocytes were available for nine patients. These samples were thawed and stimulated for 68 h. After stimulation, 10^7^ cells/sample were irradiated at room temperature with 0- or 2-Gy X-rays (200 kV, 4.0 mA, 0.5 Gy/min). After 24-h incubation, RNA was extracted with an RNeasy Mini Kit (Qiagen, http://www.qiagen.com). Biotin-labeled cRNA probes were generated starting from 2–5 μg of RNA and were hybridized to HG-U133A GeneChip arrays (Affymetrix, http://www.affymetrix.com) according to the manufacturer's recommendations. Arrays were scanned, and images were processed (MAS5) to obtain an intensity value for each oligonucleotide probe. Several parameters were considered for quality control: present calls 37%–56% (46.0 ± 0.5, average ± SEM), GAPDH 5′/3′ 0.81–2.18 (1.13 ± 0.02), and noise (RawQ) 1.04–3.56 (1.98 ± 0.06).

### Preprocessing of Microarray Data

Microarray analyses were made in R v. 2.1.1 using functions from Bioconductor v. 1.6 [34]. Additional scripts developed in house are available at http://www.medgencentre.nl/pla/index.html. For each of the 50 patients, the scanned intensities (CEL files) from the hybridizations (0 and 2 Gy) were preprocessed pairwise: background was corrected and mismatch probes were subtracted according to the MAS5 procedure, and the intensities were normalized by quantiles [35]. The multiple probe signals per gene, measured with Affymetrix microarrays, were not averaged; rather, the individual intensities were used as separate entities in a linear regression model [36]. Per gene and patient, the log_2_ signal intensities were used in the following model:

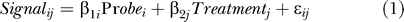
for each probe *i* and treatment *j*. The ɛ*_ij_* variables were assumed to be independent and random with mean 0 and variance σ^2^ [36]. For each gene at least 22 probe signals were measured to estimate the β coefficients. The β_1_ coefficients estimate the influence of the individual probes, and the β_2_ coefficients correspond to the treatment response of 2 Gy for each individual lymphocyte sample. Since a log base 2 was used, the β_2_ coefficient estimates can be regarded as the fold changes induced by X-irradiation. Heat maps were generated using TreeView [37]. To determine the radiation response for the subgroups, *t*-tests were performed under the null hypothesis that expression levels after 2 Gy were unchanged (β_2_ = 0). The proportion of unchanged genes was estimated using the method developed by Storey and Tibshirani [38].

### Network Analysis

The network images and accompanying analyses were generated using Ingenuity Pathways Analysis (Ingenuity Systems, http://www.ingenuity.com).

### Definition of Gene Sets

Gene sets were used as defined by the Gene Ontology (GO) Consortium [39]. Using the Bioconductor package hgu133a of May 17, 2005, on the HG-U133A array, there were 5,912 GO terms that reflected a defined cellular component, biological process, or molecular function. Of the 22,283 transcripts on the array, 8,141 were discarded because they were associated with GO terms inferred by electronic annotation, i.e., experimental proof for a biological function is lacking.

Kappa statistics were used to characterize the relationship between gene sets, providing a quantitative measure of the degree of overlap between terms sharing the same genes. For gene sets sharing large overlap (kappa > 0.8), all but one gene set were discarded. Only gene sets containing more than five members on the array were considered. After excluding gene sets based on these criteria, 1,182 gene sets remained.

### Classification Using Signature Genes

A binary clinical outcome was defined: NR or OR. Training and validation sets were repeatedly and randomly selected among 38 patients according to the method described by Michiels and co-workers [25]. The estimates of β_2_ were used as input. A resampling approach was used to randomly divide the dataset (*N =* 38) 500 times into training sets (size *n*) with *n*/2 patients of NRs and ORs, and into balanced validation sets (size *N − n*). The value of *n* was varied from ten to 34 (5–17 ORs + 5–17 NRs). In addition, we tested unbalanced random training and validation sets. We identified a molecular signature for each training set and calculated the proportion of misclassifications for each associated validation set. For each training set, the molecular signature was defined as the 50 genes with the highest correlation between β_2_ coefficients and clinical outcome as shown by Pearson's correlation coefficient. We defined two profiles (NR and OR) as vectors of the average β_2_ coefficients of these 50 signature genes in the two groups, also known as centroids. Each patient in the corresponding validation set was classified according to that patient's nearest centroid, i.e., the highest correlation between the β_2_ coefficients of the patient's signature genes and the two average profiles. Based on the 500 repeated assessments, we established the misclassification rates with 95% confidence intervals using a test of proportions.

### Classification Using Gene Sets

**Figure 1:**
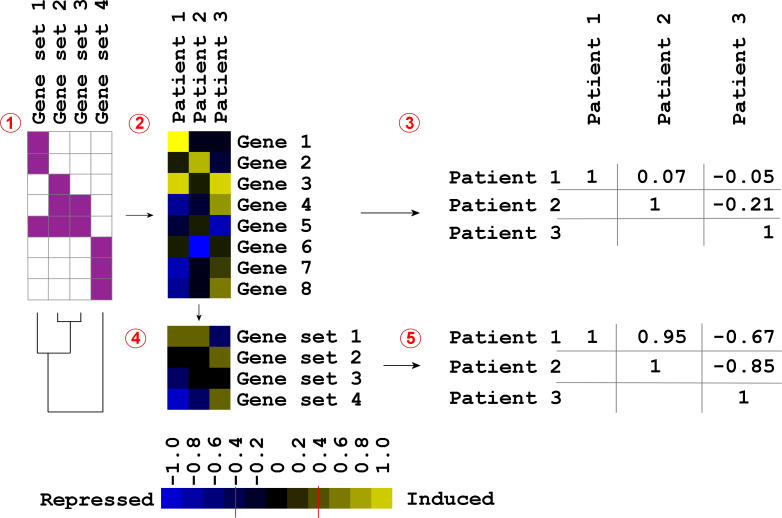
The Principle of Gene Set Classification Example of gene set analysis consisting of four gene sets, three patients, and eight genes. (1) Definition of the gene sets consisting of 2–3 genes (purple squares). Gene sets 1–3 are partially overlapping. The dendrogram shows the relationship between the gene sets identified using kappa statistics. (2) Heat map of the gene expression response per patient. (3) Pairwise correlations of all gene responses between the patients. Assuming that patient 1 and patient 3 represent different classes, patient 2 would correlate slightly better with patient 1 than with patient 3. (4) For each patient, the gene responses were combined for every gene set and visualized in a heat map. (5) Pairwise correlations of all gene sets between the patients, showing improvement in correct classification of patient 2.

The classification method for the gene sets was analogous to that for the genes. We combined the β_2_ coefficients into one value *r* (Figure 1), which was subsequently used as input in a random cross-validation procedure, as described above. The *r* values were determined as follows: a specific gene set *k* is represented by *n* genes on the array. Of these, *u* are up-regulated and *d* are down-regulated more than 1.3-fold after treatment (β_2_ < −0.4 or β_2_ > 0.4, respectively) in patient *l*. The threshold of 1.3-fold was selected since this is the average change of the most significant radio-responsive genes (Figure S1). For each gene set *k* and patient *l*, the ratio *r_kl_ =* (*u − d*)/*n* was calculated. When all genes of a gene set were up-regulated by treatment, then *r_kl_ =* 1, and, similarly, when all genes were down-regulated, then *r_kl_ =* −1. When equal numbers of genes were up- and down-regulated, then *r_kl_ =* 0.

### Calculation of Individual Risk of Late Radiation Toxicity

For each patient, the individual probability for late radiation toxicity was calculated. For the original 38 patients, with a training set size of 32 patients, the certainty of classification was calculated as the absolute difference in correlation between each patient and the NR and OR centroid, i.e., *X* = |cor(C*_i_*, C_NR_) − cor(C*_i_*, C_OR_)|, where C*_i_* is the vector of β_2_ or *r* values (for genes and gene sets, respectively) for patient *i*, and where i ∈ (1,38) and C_NR_ and C_OR_ are the centroids of 16 NR and 16 OR patients randomly selected. As the cross-validations were performed repeatedly, the tolerance interval was calculated by İ ± *K*σ where İ and σ represent the mean and the standard deviation of X and *K* = 1.93 for 95% confidence and 90% coverage. Patients were considered to be classified with certainty if the tolerance interval did not include zero.

### Classification of an Independent Set of Patients

The β_2_ and *r* values of 12 additional patients were used for validation. The genes/gene sets present in more than 20% of the 500 repeated cross-validations with a training set size of 34 patients were used to calculate the centroid of the β_2_ and *r* values in the OR and NR groups. The samples were classified according to the nearest centroid, as previously.

The individual certainty of classification was calculated as before, but patients were considered to be confidently classified if the difference in correlations was larger than 0.2 (the smallest difference in correlations in the training set resulting in a certain classification).

## Results

From a cohort of 800 patients treated by radiotherapy for prostate cancer, 21 ORs with grade III toxicity and 17 NRs with grade 0 toxicity were selected. The OR and NR patient groups were comparable in age, primary tumor stage, hormone use, and radiation dose and volume (Table S1). No significant group differences were found in the absolute number of white blood cells and the percentages of monocytes, lymphocytes, and granulocytes between a control group [40] and a cohort of 12 patients (*n =* 6 ORs, *n =* 6 NRs) as determined by flow cytometry.

As a first attempt towards distinguishing the NR and OR groups, the gene expression response of lymphocytes to X-ray treatment was investigated. The treatment effect (β_2_) on each gene and patient was identified by linear regression, and the radio-responsive genes in each patient group were identified by statistical testing (Figure S1A–S1D). In both groups, irradiation of stimulated lymphocytes led to the induction (*p* < 0.001) of well-known radio-responsive genes [20–22] such as *CDKN1A*, *GADD45A*, *FAS, DDB2,* and *XPC* (Figure S2A and S2B). Considerable patient-to-patient variation in expression was detected in the most significantly radio-responsive genes. However, this variability was not linked to patients' radiation toxicity. Grouping the most radio-responsive genes by *k*-means (*k* = 2) clustering [41] showed no correlation to toxicity status (OR and NR, Figure S2C).

**Figure 2:**
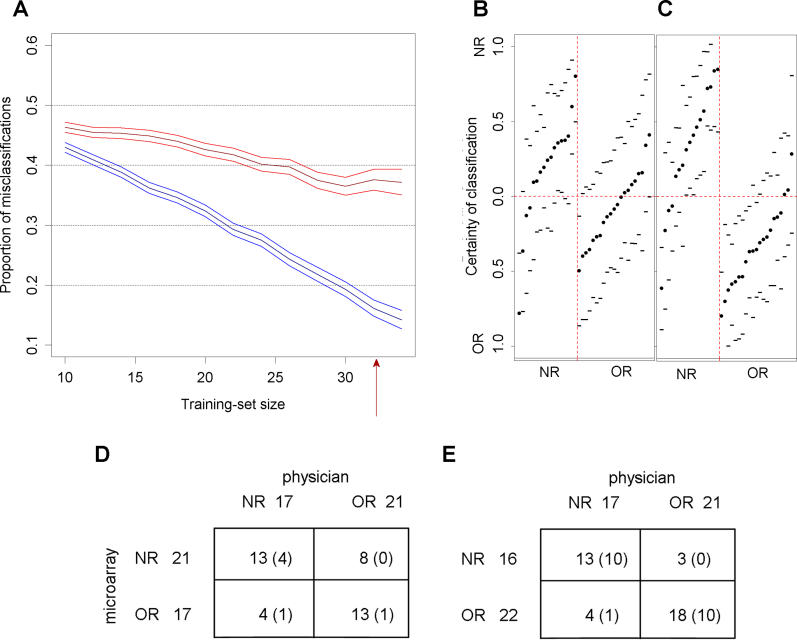
Improved Patient Classification Using Functionally Related Gene Sets (A) Gene classification (in red) and gene set classification (in blue), following the strategy of Michiels et al. [25], with 95% confidence intervals for the test of proportions. The minimal misclassification rate was 37% ± 2% with gene classification and 14% ± 2% with gene set classification. (B and C) The certainty of microarray classification for each patient was calculated based on (B) genes or (C) functionally related gene sets. The certainty was calculated at the training set size of 32 patients (red arrow in [A]). (D and E) Contingency tables summarizing the concordance between the physician and microarray classifications using (D) genes and (E) gene sets. Numbers of patients classified with certainty (cases where the tolerance limit does not include zero) are in parentheses.

To find a molecular signature for the development of late toxicity, a cross-validated classifier was constructed within the patient data set using the β_2_ coefficients as input. We compared the results of a random validation method based on a conventional classification using separate gene expression levels with those of a classification based on functionally related gene sets (Figure 2). With the former, the maximum proportion of correct classification using the 50 individual genes with the highest correlation to responder status was 0.63 ± 0.02 (Figure 2A). There was a clear trend towards better classification with increasing training set size. We estimated the certainty of classification of each patient (Figure 2B and 2D) using the cross-validation results at a training set size of 32 patients (the largest training set size for which the associated validation set still contained an NR patient). The certainty (including tolerance limits of 95% confidence and 90% coverage) was calculated as stated in the Methods. Only six patients, whereof one was misclassified, had tolerance limits not including zero.

**Figure 3:**
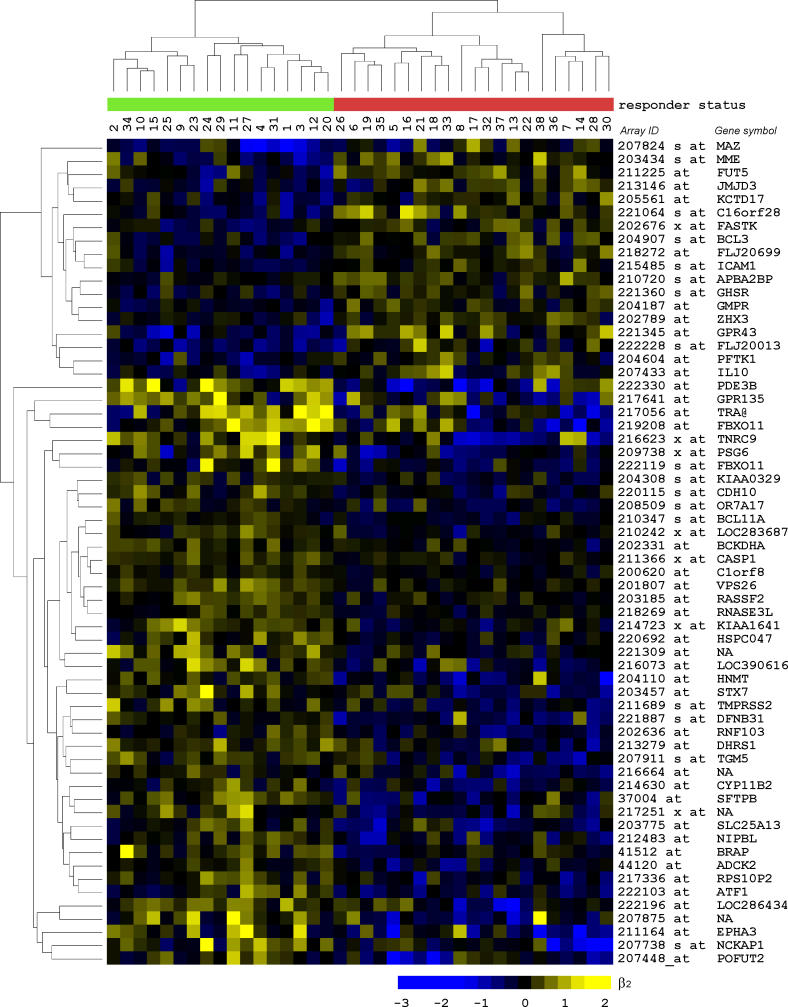
Expression Profiles of Classifying Genes Heat map of β_2_ values of 62 genes that were present in more than 20% of the 500 repeated assessments with 34 patients in the training set of the classifier. These discriminating genes were used in a supervised two-dimensional hierarchical clustering of NRs (green) and ORs (red) based on the β_2_ values representing the radiation response.

**Figure 4:**
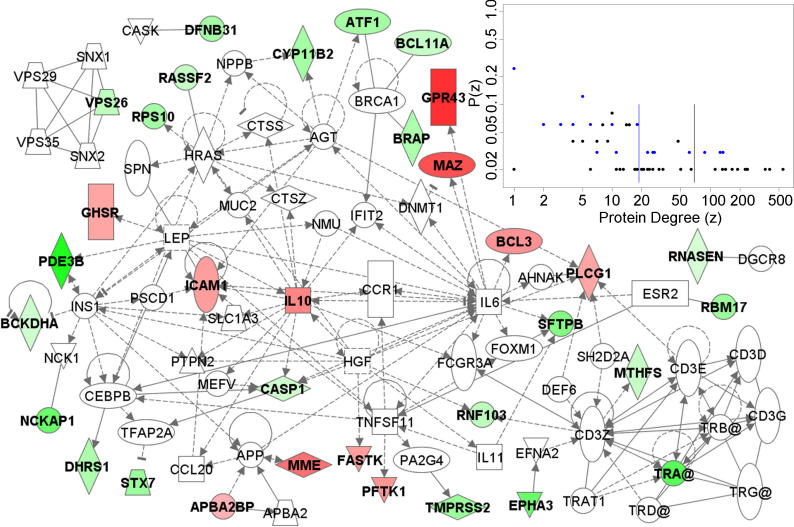
The Interactions of Proteins Representing the Gene Classifier Of the gene products most frequently present in the gene classifier, 33 proteins are present in the Ingenuity database. These are represented by colored symbols (green symbols indicate proteins that have higher induction after irradiation in NRs, and red symbols indicate proteins that have higher induction in ORs). The intensity of the colors indicates the difference between the groups in the magnitude of induction. The connecting proteins are represented by empty symbols. Only three of the colored proteins are not directly or indirectly linked through a connecting protein. Inset: The degree distribution of the proteins in the sub-network. For each protein we calculated the number of interactions in the total human interaction network (protein degree, *z*) and plotted it against the proportion of each protein degree, *P*(*z*). Vertical blue and black lines indicate the average protein degree, showing that the classifier proteins (blue) and the connecting proteins (black) represent two separate populations (*p* < 0.001, Wilcoxon test).

We found 62 genes to be present in more than 20% of the 500 assessments performed (Figure 3). To investigate whether these genes function in common pathways, we studied their connectivity by Ingenuity pathway analysis. Ten pathways with more than five pathway members—including small molecule biochemistry, cell morphology, cancer, cell death, and immune response—were significantly (*p* < 0.01) enriched among these classifying genes. When relating the classifying genes by the interactions of their gene products, a highly connected network emerged (Figure 4). For the proteins in this sub-network of the human interactome, we calculated the number of interaction partners (protein degree). Interestingly, the classifying proteins had significantly (*p* < 0.001, Wilcoxon test) less direct connections than the connecting proteins, with means of 18.7 and 68.6 interactions, respectively. This suggests that the classifying proteins interact with, but are not themselves, “master regulators” (highly connected hubs).

We next used gene sets based on function or cellular colocalization reported in publicly available databases to distinguish the NR and OR groups (Figure 1). For each gene set, the combined fractions of induced and repressed genes in each patient (*r* value, see Methods) were used as input in the random validation classifier. Genes whose expression changed more than 1.3-fold were selected as induced or repressed. This rather low threshold was used as the expression of many genes is affected by X-rays to a moderate extent (Figure S1). Gene set classification substantially improved the classification result, increasing the proportion of correct classifications to 0.86 ± 0.02 (Figure 2A). The gene set classifier performed better than the gene classifier for all training set sizes.

Additionally, we tested the stability of our classification results by varying the number of both genes and gene sets in the classifiers between 20 and 70. Classification performance—especially that of the gene set classifier—remained stable and independent of the number of gene sets used as input in the classifier (Figure S3B and S3D). Also, the classifiers were tested based on totally random attribution to training and validation sets (as opposed to the balanced attribution of equal numbers of NR and OR patients in the training set). Third, for better comparison between gene and gene set classification, gene classification was performed on β_2_ coefficients for genes that were filtered for annotation and within a reasonably sized gene set size (5< *n* <500). In all tests gene set classification gave better classification than individual genes. Classification results of all tests were largely within the previously determined 95% confidence intervals (Figure S3).

To investigate whether the estimate of classification certainty of each patient was also improved by gene set classification, we dissected the cross-validation results at a training set size of 32 patients (Figure 2C and 2E). The certainty of classification was indeed predicted with higher accuracy with the gene set classifier. For 31 out of the 38 patients, toxicity status was correctly classified on an individual basis, and 21 of the 38 patients were classified with certainty (had tolerance intervals not including zero). Only one of these patients was misclassified.

**Figure 5:**
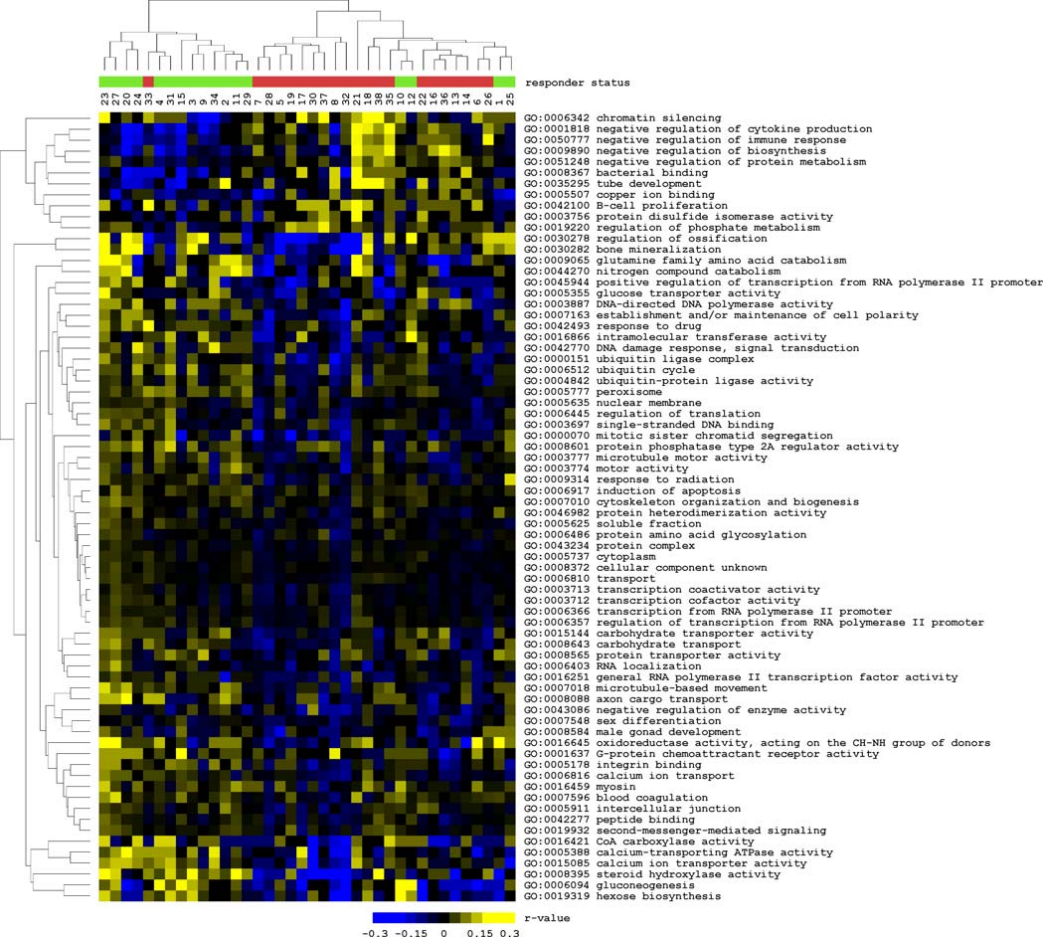
Expression Profiles of Classifying Gene Sets Heat map of *r* values of 72 gene sets that were present in more than 20% of the 500 repeated assessments with 34 patients in the training set of the classifier. These discriminating gene sets were used in a supervised two-dimensional hierarchical clustering of NRs (green) and ORs (red) based on the *r* values The threshold for being affected was set at |β_2_| = 0.4.

Frequently occurring gene sets among the gene set classifiers may provide insight into the pathways that are differentially regulated between the two patient groups. Examining the classifying gene sets at a training set size of 34 patients, 72 were found in more than 20% of the 500 repeated assessments. Among the most frequently occurring gene sets are gene sets engaged in protein metabolism and ubiquitination, development, stress signaling, and apoptosis (Figure 5). The apoptotic response, represented by the gene set “induction of apoptosis,” is more pronounced in NR group than the OR group.

To test the reproducibility of classification, additional blood samples were taken at least 1 y after the original sampling from four patients. The gene set classifier reproduced the predictions for responder status of all four patients, whereas the gene classifier produced a different outcome for half of the samples.

**Figure 6:**
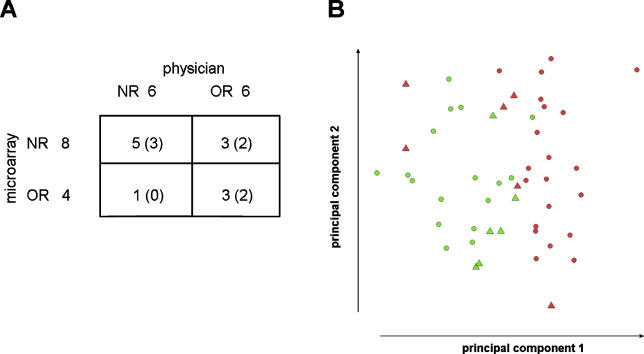
Validation of the Gene Set Classification with an Independent Patient Set (A) Contingency table of the physician and microarray classification of 12 additional patients. The 72 most discriminating gene sets in the training set were used to predict responder status. Numbers of patients classified with certainty are in parentheses. (B) A principal components analysis plot of the two principal components separating the NRs (green) from the ORs (red). Circles represent the 38 patients of the original training set, and triangles represent the 12 patients of the independent validation set.

A small independent set of 12 additional patients (Table S2) was used to validate the gene and gene set classifiers. Unfortunately, we had to adjust the experimental protocol to some extent, since only three patients could be recruited to provide fresh lymphocytes. From nine patients, limited amounts of frozen lymphocytes were available, and after applying a modified protocol, enough RNA was obtained for microarray hybridization. Gene set classification predicted a responder status of eight of these 12 new patients identical to that recorded by the physician (Figure 6A). Seven patients were classified with certainty, whereof five classifications were correct. To get a visible impression for all patients of the performance of the classifying gene sets, a principal component analysis was carried out, which revealed two main clusters of NRs and ORs (Figure 6B). The gene classifier did not allow classification of the validation set (Figure S4).

## Discussion

This study shows that analysis of gene expression profiles can be greatly improved by considering the joint behavior of functionally related genes. With this approach, we were able to substantially improve correct classification of NRs and ORs among prostate cancer patients that had received radiotherapy. Our findings also provide support for the existence of a genetic component to late radiation toxicity. A clear association was found between the development of late normal tissue reactions following radiotherapy and the gene expression responses of patients' ex vivo irradiated lymphocytes. Patient toxicity status may therefore be related to interindividual variability in the response to radiation-induced DNA damage. The clinical trait of normal tissue radiation toxicity may result from a combination of polymorphisms in DNA-damage-responsive genes [42] that influence the expression of multiple functionally connected genes. Interestingly, the apoptotic response appeared to be more pronounced in patients who did not develop toxicity, similar to the results of Barber et al. [14]. It is tempting to speculate based on our findings that a more pronounced apoptotic response protects patients against developing late radiation toxicity.

Our results suggest that interpatient variability in late radiation toxicity relates to individual differences in downstream targets of cellular pathways, in addition to apoptosis, involved in protein metabolism, ubiquitination, and stress signaling. These downstream targets are less conserved than hubs [43] and therefore are plausible sources of interindividual variation. However, no single gene or functionally related set of genes was found that by itself correlated perfectly with the observed clinical radiation toxicity. This finding is consistent with results from Rieger and co-workers [19] that suggest a connection between acute toxicity and alterations in six main cellular processes: DNA repair, stress response, cell cycle, ubiquitination, apoptosis, and RNA processing. Although similar cellular processes seem to be involved in our study, there is little overlap between the two studies in the individual genes identified. This is not unexpected given that acute and late radiation toxicity are distinct, albeit likely related, phenomena. This finding, however, provides additional support for the hypothesis that individual susceptibility to late radiation toxicity is substantially determined by genetic predisposition. On the other hand, our results do not exclude the additional role of other factors such as age, radiation dose and volume, and comorbidity. Stochastic effects also contribute to the uncertainty of classification [6].

There are numerous choices to be made when performing gene set classification. A variety of properties can be used to group genes into sets. While we used GO terms as the basis for gene set definition, an alternative approach is to group genes based on protein–protein interactions or transcription factor binding activities. Furthermore, we had to choose a β_2_ threshold for the calculation of *r* values. In general, it is important that threshold settings allow generous selection of affected genes [44]. The β_2_ values for the most significantly changed genes (Benjamini-Hochberg false discovery rate of 5%) were 0.36 and 0.39 for the NR and OR groups, respectively (Figure S1). This corresponds to a fold change of 1.3. Therefore a threshold of |β_2_| = 0.4 was chosen for the calculation of the *r* values for gene set classification. We also had to select a method for combining the expressions of the multiple genes belonging to a gene set into a single value. Segal and co-workers recently proposed using the significance of modules for this purpose [28]. In our case, because the radiation responses of the classifying genes were of opposite direction (up-regulated in NR and down-regulated in OR, or vice versa; Figure 3), we chose to calculate for each patient and gene set a single *r* value by subtracting the number of down-regulated genes from the number of up-regulated genes and dividing that by the total number of genes in that gene set (*r* = [*u − d*]/*n*).

Although the performance of our gene set classifier was significantly better than that of the gene classifier, the discriminative power and reliability of the latter were still significantly better than in most studies using microarrays to predict cancer outcome [25]. We deliberately chose to use parameters settings (i.e., 50 top features, 500 assessments) identical to those used by Michiels et al. [25] when reanalyzing several cancer studies. Two striking differences of the present analysis were observed: our misclassification rates were lower, while our confidence intervals were narrower. We attribute these differences to two factors. First, we did not study basal expression but instead treatment response, where each irradiated sample was compared to the unirradiated control of the same patient. Second, we studied normal tissue instead of cancer cells, which are notorious for their genetic heterogeneity, instability, and variability. A consequence of adapting a classifier according to Michiels et al. is the calculation of confidence instead of tolerance intervals. The obtained narrow 95% confidence limits are not very informative as they result from 500 iterations. For example, with an independent validation set of only 12 samples, a 14% misclassification rate would lead to a much wider 95% confidence interval, ranging from 0% to 35%. The 33% misclassification we found for our validation set is within this range. The adjustments we had to make in the experimental protocol for part of the validation set may have caused the misclassification rate to be on the higher side of the confidence interval. This issue highlights the need for standardized procedures [45]. Furthermore, these misclassification rates are population-based, while in the end it is the certainty of correct classification of individual patients that is of clinical value.

With our classification method, classification with certainty was achieved for 55% of the patients, a percentage that needs to be improved before clinical application of this methodology. Several explanations exist for the failure to classify 45% of the patients with certainty. First, the “correctness” of patient classification by the physician is crucial, as intentional switching of patient labels drastically affects misclassification rates (data not shown). For some misclassified patients our analysis suggests that they genetically belong to another group than suggested by their clinical symptoms. A subset of patients might show symptoms resembling radiation toxicity, but unlinked to the radiation therapy. Second, modifiers of toxicity not yet identified could affect the certainty of classification. These results indicate that this gene set classifier will be suitable for only approximately half of the extreme responders.

Nevertheless, caution should be taken in regarding the identified gene sets as unique indicators for late radiation toxicity. We do not envision that we have captured the whole spectrum of interindividual variation in gene expression with this rather limited study of 50 patients. A study of more patients is likely to reveal subgroups of genetically determined extreme responders. Also, due to the retrospective nature of this case-control study, we cannot exclude the possibility that our findings reflect the genetic consequences of, rather than the basis for, late radiation toxicity. However, the fact that we based our classification on lymphocytes' ex vivo radiation response rather than basal levels of gene expression argues against this possibility. The current retrospective study has paved the way for the larger prospective validation study needed to clarify this matter. Such a study is essential before these results can be translated into the clinic. To maximize the chance of finding measurable effects at the gene expression level, we selected homogeneous groups of patients from the two extremes of a wide scale of clinical radiation toxicity. While additional work is needed to investigate the applicability of our findings to more moderate levels of radiation toxicity, the present findings support the notion that in the future we may be able to attain the desired outcome of predicting severe late radiation toxicity prior to radiotherapy.

## Supporting Information

Alternative Language Abstract S1 Translation of Abstract into Swedish by Author J. P. S. (21 KB DOC)

Alternative Language Abstract S2 Translation of Abstract into Dutch by Author B. K. (21 KB DOC)

Figure S1 Characteristics of the Radiation Response in NR and OR Patients (A and B) Density distribution of β_2 _values of genes after applying the model: Signal = β_1_ Probe + β_2_ Treatment + ɛ on the background-corrected and normalized log_2_ signal intensities of each patient and gene. A 2-fold change (β_2_ = −1 or β_2_ = 1) is commonly used as a threshold to characterize a gene expression response. In these patient groups, expression of 4.0% (NR group) and 4.4% (OR group) of the genes was changed more than 2-fold (β_2_ < −1 or β_2_ > 1, red area).
(C and D) Density distribution of *p*-values for genes being radio-responsive after *t*-testing of the subgroups of NRs and ORs. The estimated proportion of unchanged genes is indicated by a red line.
(E and F) From the above it was calculated that the proportion of radio-responsive genes was 24% and 21% for NRs and ORs, respectively. In order to find a more relevant threshold than a 2-fold change, we determined the mean of the β_2_ values for the most significantly changed genes (Benjamini-Hochberg false discovery rate of 5%), which was 0.36 and 0.39 for NRs and ORs, respectively (red lines). This corresponds to a fold change of 1.3. This threshold was used for the calculation of the *r* values needed for the gene set classification.
(A,C, and E) show the NR and (B,D and F) the OR.
(164 KB TIF)

Figure S2 Heat Maps of β_2_ Values of Radio-Responsive Genes (A and B) The 100 most significantly up- and down-regulated genes for each patient group as determined by -testing of the β_2_ values for NRs (A) and ORs (B).
(C) The combination of the genes in (A) and (B) yields 162 radiation responsive genes. A *k*-means clustering separated the patients into two groups (top bar, grey versus black), which were unrelated (Pearson's correlation coefficient = 0.03) to the responder status (secondmost top bar, green [NRs] versus red [ORs]).
(398 KB TIF)

Figure S3 Variation of Selection Criteria for Classification As input we varied the number of genes/gene sets (20 ≤ *N* ≤ 150) with the highest correlation between β_2_/*r* values) and responder status. (A, C, and E) show the results from the genes and (B and D) show the results from the gene sets.
(A and B) Balanced classifier (equal numbers of NRs and ORs in the training sets).
(C and D) Unbalanced classifier (random attribution of NRs and ORs to the training sets).
(E) Gene classifier after filtering the genes for having annotation and belonging to a gene set with 5–500 members.
(213 KB TIF)

Figure S4 Validation of the Gene Classification on an Independent Patient Set (A) The 62 most discriminating genes in the training set were used to predict responder status for 12 additional patients.
(B) A principal components analysis plot of the two principal components separating the NRs (green) from the ORs (red). Circles represent the 38 patients of the original training set, and triangles represent the 12 patients of the independent validation set.
(69 KB TIF)

Table S1 Patient Characteristics of NRs and ORs (54 KB DOC)

Table S2 Patient Characteristics of Validation Set (*n* = 12) (56 KB DOC)

### Accession Numbers

The intensity (CEL) files produced in this study were deposited in ArrayExpress (http://www.ebi.ac.uk/arrayexpress) with accession number E-TABM-90. The GeneID (http://www.ncbi.nlm.nih.gov/entrez/query.fcgi?CMD=search&DB= gene) accession numbers for the genes discussed in this paper are *CDKN1A* (1026), *DDB2* (1643), *FAS* (355)*, GADD45A* (1647), and *XPC* (7508).

## Abbreviations

GO: Gene Ontology
NR: non-responder
OR: over-responder
