# Supplementary material for: Analysis of Gene Expression Using Gene Sets Discriminates Cancer Patients with and without Late Radiation Toxicity
Source: PLoS Med. 2006 Oct 31;3(10):e422. doi: 10.1371/journal.pmed.0030422 (PMC1626552; doi:10.1371/journal.pmed.0030422)
Supplement: Alternative Language Abstract S1 — (21 KB DOC) [file pmed.0030422.sd001.doc]

### Gensetanalys av genuttryck diskriminerar mellan cancerpatienter med och utan sen stråltoxicitet

**Bakgrund**

Strålning utgör en effektiv anti-cancerbehandling men leder till allvarlig sen stråltoxicitet för 5-10% av de behandlade patienterna. Förutsatt att genetiska faktorer påverkar denna risk, hypotiserar vi att cellsvaret efter bestrålning hos normal vävnad skulle kunna diskriminera mellan patienter som utvecklat sen stråltoxicitet eller inte.

**Metod**

Prostatacancerpatienter utan bevis för cancer 2 år efter kurativ strålbehandling rekryterades till studien. Blodprov från 21 patienter med allvarliga sena strålkomplikationer och 17 patienter utan symptom samlades in. Stimulerade perifera lymfocyter röntgenbestrålades med 0 eller 2 Gy. Efter 24 timmar isolerades RNA och gene uttryck analyserades med microarrays för att användas för klassificering av patienterna. Klassificeringen utfördes antingen på bas av sepatata gener eller, för att förbättra styrkan, på bas av genset bestående av gener grupperade efter funktion eller gemensam cellulär lokalisering.

**Huvudsakliga fynd**

Bestrålning förändrade uttryck av strålresponderande gener i båda grupperna. Responsen var variabel över patienterna och uttryck av de mest signifikant responderande generna var okorrelerat till strålkänslighet.

Klassificeraren baserad på strålresponsen hos individuella gener klassificerade 63% av patienterna korrekt. Klassificeraren baserad på förändrade genset förbättrade den korrekta klassificeringen till 86%, även om endast 21/38 patienter kunde klassificeras med säkerhet. Majoriteten av de diskriminerande generna och genseten berörde ubiquitin-, apoptos- och stresssignalerings-nätverken. Det apoptotiska svaret föreföll mer uttalat hos patienter som inte utvecklat toxicitet. I en oberoende grupp av 12 patienter kunde toxicitet förutsägas korrekt i 8 fall av gensetklassificeraren.

**Slutsatser**

Genom profilering av genutryck åstadkoms en separering av patientgrupper med eller utan utveckling av sen stråltoxicitet. Den diskriminerande styrkan förbättrades genom användning av funtionellt eller strukturellt besläktade genset. Å andra sidan, för endast hälften av patienterna kunde toxicitetsstatus bestämmas med säkerhet. Den här studien är ett steg mot att kunna förutsäga individuell benägenhet för att utveckla sen stråltoxicitet.
