# Supplementary material for: Analysis of Gene Expression Using Gene Sets Discriminates Cancer Patients with and without Late Radiation Toxicity
Source: PLoS Med. 2006 Oct 31;3(10):e422. doi: 10.1371/journal.pmed.0030422 (PMC1626552; doi:10.1371/journal.pmed.0030422)
Supplement: Alternative Language Abstract S2 — (21 KB DOC) [file pmed.0030422.sd002.doc]

### Analyse van genexpressie met behulp van genensets onderscheidt kankerpatiënten met en zonder late toxiciteit na bestraling

**Achtergrond**

Bestraling is een effectieve behandeling tegen kanker, maar leidt in 5-10% van de patiënten tot ernstige late toxiciteit. In de veronderstelling dat genetische gevoeligheid dit risico beinvloedt, postuleerden wij dat patiënten met en zonder late stralingstoxiciteit van elkaar onderscheiden zouden kunnen worden door de cellulaire response op bestraling van normaal weefsel te meten

**Methodologie**

In deze studie werden prostaatkankerpatiënten opgenomen die 2 jaar na curatieve bestraling tumorvrij waren. Van 21 patiënten met ernstige late complicaties na bestraling en 17 patiënten zonder symptomen werden bloedmonsters verzameld. Gestimuleerde perifere lymfocyten werden röntgen bestraald met een dosis van 0 en 2 Gy. De bestralingsrespons werd na 24 uur met behulp van gen expressie profilering geanalyseerd en gebruikt voor classificatie van de patiënten. De classificatie werd uitgevoerd ofwel op basis van expressie van individuele genen ofwel, om de mate van de classificatie te versterken, op basis van sets van genen gegroepeerd naar functie of cellulair co-lokalisatie.

**Resultaten**

Röntgen bestraling veranderde de expressie van stralingsgevoelige genen in beide groepen. Deze response was variabel in individuele patiënten en expressie van de meest significant veranderde genen was niet gecorreleerd aan toxiciteit na bestraling. De “classifier” op basis van de bestralingsresponse van individuele genen classificeerde 63% van de patiënten correct. De classificatie gebaseerd op veranderde sets van genen verhoogde deze correcte classificatie tot 86%, alhoewel slechts 21 van de 38 patiënten (55%) met grote zekerheid werden geclassificeerd. Het beste onderscheid kon worden gemaakt met behulp van genen en sets van genen die tot de ubiquitine, apoptose of stress signaal netwerken behoorden. De apoptose response was sterker in de patiënten die geen toxiciteit ontwikkelden. In een onafhankelijke groep van 12 patiënten werd de toxiciteit voor 8 patiënten juist voorspeld met behulp van de classifier van sets van genen.

**Conclusies**

Gen expressie profilering kan groepen van patiënten met en zonder late toxiciteit na bestraling van elkaar onderscheiden. Het gebruik van functionele of structurele sets van genen verhoogt de mate van discriminatie. Hoewel de classifier voor de response op individueel niveau nog verbeterd moet worden, is deze studie een belangrijke stap vooruit om de gevoeligheid voor late toxiciteit na bestraling te voorspellen.
