# Supplementary figures and images for: Analysis of Gene Expression Using Gene Sets Discriminates Cancer Patients with and without Late Radiation Toxicity
Source: PLoS Med. 2006 Oct 31;3(10):e422. doi: 10.1371/journal.pmed.0030422 (PMC1626552; doi:10.1371/journal.pmed.0030422)

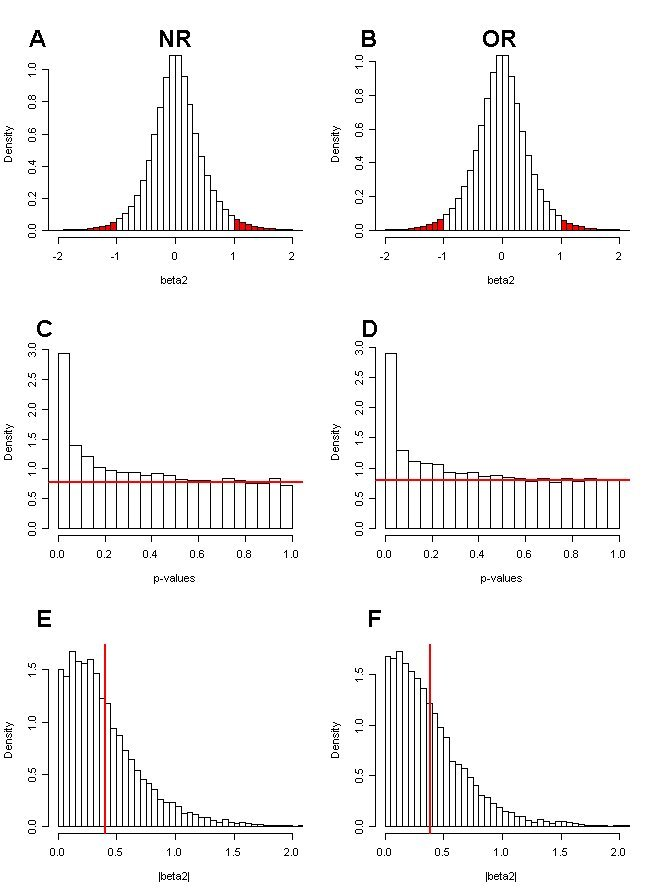

Supplement: Figure S1 — (A and B) Density distribution of β2 values of genes after applying the model: Signal = β1 Probe + β2 Treatment + ɛ on the background-corrected and normalized log2 signal intensities of each patient and gene. A 2-fold change (β2 = −1 or β2 = 1) is commonly used as a threshold to characterize a gene expression response. In these patient groups, expression of 4.0% (NR group) and 4.4% (OR group) of the genes was changed more than 2-fold (β2 < −1 or β2 > 1, red area). (C and D) Density distribution of p-values for genes being radio-responsive after t-testing of the subgroups of NRs and ORs. The estimated proportion of unchanged genes is indicated by a red line. (E and F) From the above it was calculated that the proportion of radio-responsive genes was 24% and 21% for NRs and ORs, respectively. In order to find a more relevant threshold than a 2-fold change, we determined the mean of the β2 values for the most significantly changed genes (Benjamini-Hochberg false discovery rate of 5%), which was 0.36 and 0.39 for NRs and ORs, respectively (red lines). This corresponds to a fold change of 1.3. This threshold was used for the calculation of the r values needed for the gene set classification. (A,C, and E) show the NR and (B,D and F) the OR. (164 KB TIF) [file pmed.0030422.sg001.tif]

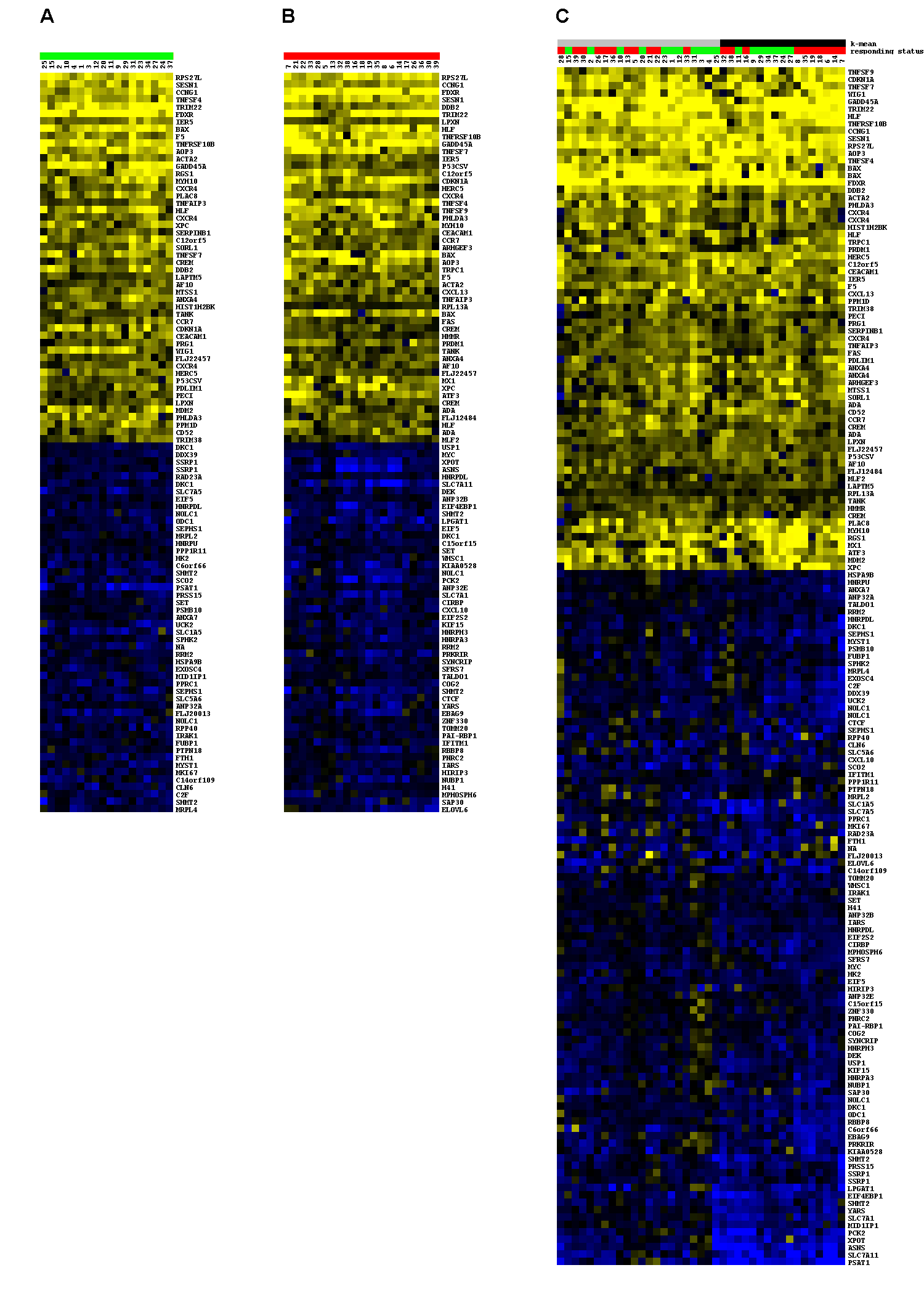

Supplement: Figure S2 — (A and B) The 100 most significantly up- and down-regulated genes for each patient group as determined by -testing of the β2 values for NRs (A) and ORs (B). (C) The combination of the genes in (A) and (B) yields 162 radiation responsive genes. A k-means clustering separated the patients into two groups (top bar, grey versus black), which were unrelated (Pearson's correlation coefficient = 0.03) to the responder status (secondmost top bar, green [NRs] versus red [ORs]). (398 KB TIF) [file pmed.0030422.sg002.tif]

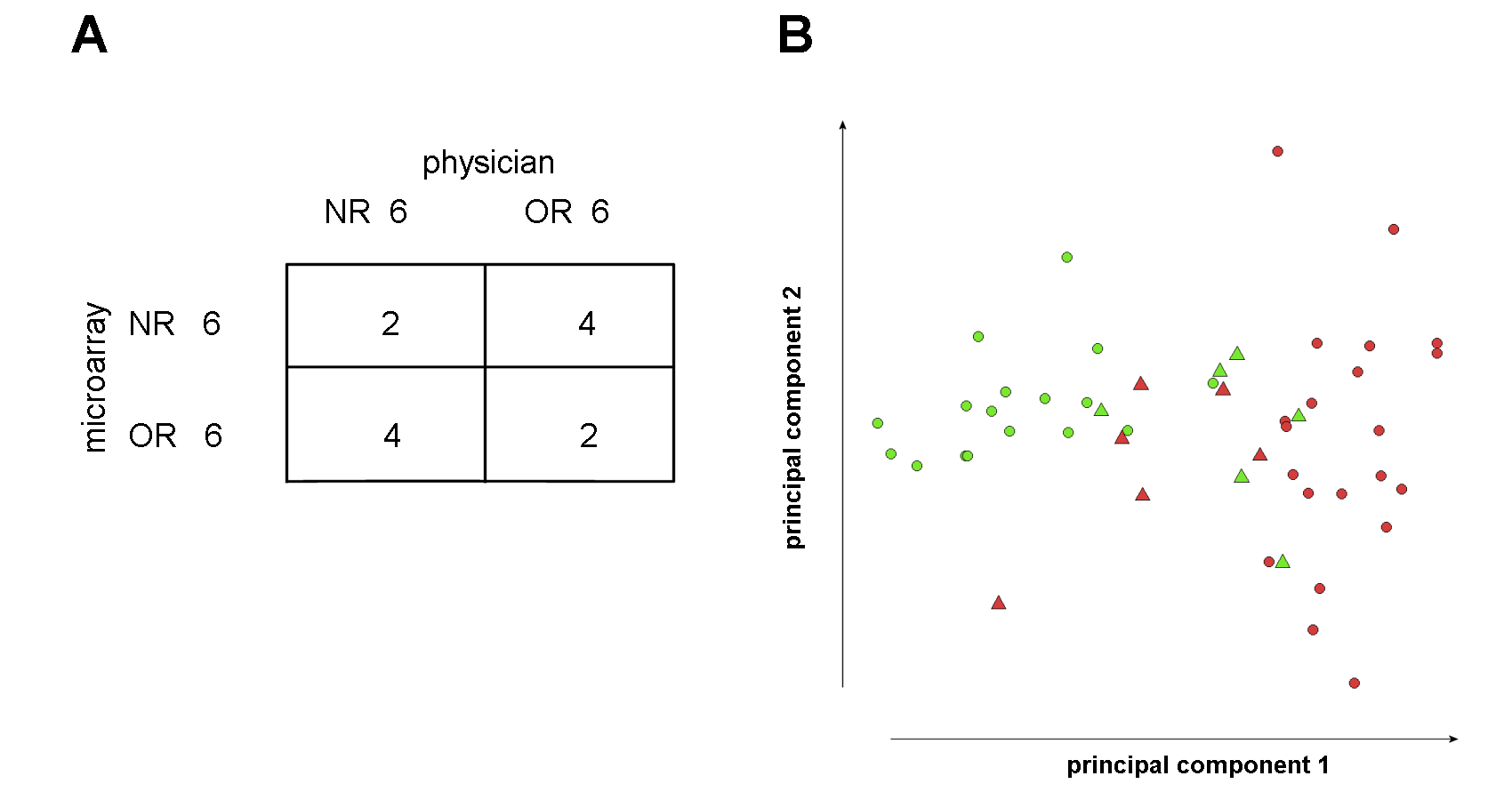

Supplement: Figure S4 — (A) The 62 most discriminating genes in the training set were used to predict responder status for 12 additional patients. (B) A principal components analysis plot of the two principal components separating the NRs (green) from the ORs (red). Circles represent the 38 patients of the original training set, and triangles represent the 12 patients of the independent validation set. (69 KB TIF) [file pmed.0030422.sg004.tif]
