# Supplementary material for: Analysis of Gene Expression Using Gene Sets Discriminates Cancer Patients with and without Late Radiation Toxicity
Source: PLoS Med. 2006 Oct 31;3(10):e422. doi: 10.1371/journal.pmed.0030422 (PMC1626552; doi:10.1371/journal.pmed.0030422)
Supplement: Table S1 — (54 KB DOC) [file pmed.0030422.st001.doc]

**Table S1.** Patient Characteristics of NRs and ORs

| Variable | Subcategory | NRs (*n* = 17) | | | | ORs (*n* = 21) | | | | *p*-Value |
| --- | --- | --- | --- | --- | --- | --- | --- | --- | --- | --- |
|  |  | *n* | Range | Mean Value | 95% C.I. | *n* | Range | Mean Value | 95% C.I. |  |
| Age |  | 17 | 57–86 | 75.7 | 71.7–79.8 | 21 | 51–85 | 73.8 | 70.2–77.3 | 0.50 tt |
| PSA^a^ before radiotherapy |  | 17 | 5.3–70.0 | 21.0 | 13.0–28.9 | 21 | 2.0–43.8 | 14.4 | 9.2–19.6 | 0.12 tt |
| T-classification^b^ | T1 | 2 |  |  |  | 5 |  |  |  | 0.35 χ^2^ |
|  | T2 | 2 |  |  |  | 3 |  |  |  |  |
|  | T3 | 11 |  |  |  | 13 |  |  |  |  |
|  | T4 | 2 |  |  |  | 0 |  |  |  |  |
| Radiotherapy | Local only (70 Gy) | 6 |  |  |  | 10 |  |  |  | 0.49 χ^2^ |
|  | Locoregional (40 Gy + 30 Gy or 50 Gy + 20 Gy) | 11 |  |  |  | 11 |  |  |  |  |
| Irradiated volume (local fields in cc) |  | 17 | 414–1,231 | 749 | 622–878 | 20 | 489–1234 | 758 | 669–847 | 0.46 tt |
| Irradiated volume (pelvic fields in cc) |  | 10 | 994–3,054 | 2,118 | 1,795–2,612 | 11 | 1,694–2,860 | 2,283 | 2,003–2,564 | 0.91 tt |
| Hormonal therapy | No | 4 |  |  |  | 7 |  |  |  | 0.44 χ^2^ |
|  | Yes | 13 |  |  |  | 14 |  |  |  |  |
| Duration of follow-up (years)^c^ |  | 17 | 2.1–10.1 | 5.5 | 4.3–6.7 | 21 | 2.0–12.7 | 4.6 | 3.5–5.8 | 0.29 tt |

^a^Prostate-specific antigen, an important biochemical marker for prostate cancer; normal value < 4 ng/ml.

^b^T-classification: clinical classification for primary tumor extension; T1, non-palpable; T2, palpable, within prostate; T3, extension beyond prostate; T4, invasion of adjacent organs.

^c^Duration of follow-up is time between last date of radiotherapy and assessment of toxicity/gene expression profiling.

χ^2^, Chi-square test; C. I., confidence interval; tt, independent sample *t*-test.
