# Supplementary material for: Analysis of Gene Expression Using Gene Sets Discriminates Cancer Patients with and without Late Radiation Toxicity
Source: PLoS Med. 2006 Oct 31;3(10):e422. doi: 10.1371/journal.pmed.0030422 (PMC1626552; doi:10.1371/journal.pmed.0030422)
Supplement: Table S2 — (56 KB DOC) [file pmed.0030422.st002.doc]

**Table S2.** Patient Characteristics of Validation Set (*n* = 12)

| Variable | Subcategory | NRs (*n* = 6) | | | | ORs (*n* = 6) | | | | *p*-Value |
| --- | --- | --- | --- | --- | --- | --- | --- | --- | --- | --- |
|  |  | *n* | Range | Mean Value | 95% C.I. | *n* | Range | Mean Value | 95% C.I. |  |
| Age |  | 6 | 58–78.2 | 68.7 | 59.4–77.9 | 6 | 62.4–79.3 | 72.5 | 65.7–79.4 | 0.40 tt |
| PSA^a^ before radiotherapy |  | 6 | 4–20 | 9.1 | 3–15 | 6 | 8–14 | 10.6 | 8–13 | 0.57 tt |
| T-classification^b^ | T1 | 0 |  |  |  | 0 |  |  |  | 0.21 χ^2^ |
|  | T2 | 2 |  |  |  | 0 |  |  |  |  |
|  | T3 | 4 |  |  |  | 5 |  |  |  |  |
|  | T4 | 0 |  |  |  | 1 |  |  |  |  |
| Radiotherapy | Local only (70 Gy) | 3 |  |  |  | 1 |  |  |  | 0.22 χ^2^ |
|  | Locoregional (40 Gy + 30 Gy or 50 Gy + 20 Gy) | 3 |  |  |  | 5 |  |  |  |  |
| Irradiated volume (local fields in cc) |  | 6 | 499–973 | 728 | 510–946 | 6 | 634–1,053 | 823 | 643–1,003 | 0.40 tt |
| Irradiated volume (pelvic fields in cc) |  | 3 |  |  |  | 1 |  |  |  | —^c^ |
| Hormonal therapy | No | 3 |  |  |  | 1 |  |  |  | 0.22 χ^2^ |
|  | Yes | 3 |  |  |  | 5 |  |  |  |  |
| Duration of follow-up (years)^d^ |  | 6 | 2.0–6.5 | 3.4 | 1.5–5.1 | 6 | 1.9–12.0 | 4.3 | 1.5–8.3 | 0.60 tt |

^a^Prostate-specific antigen, an important biochemical marker for prostate cancer; normal value < 4 ng/ml.

^b^T-classification: clinical classification for primary tumor extension; T1, non-palpable; T2, palpable, within prostate; T3, extension beyond prostate; T4, invasion of adjacent organs.

^c^Sample size too small.

^d^Duration of follow-up is time between last date of radiotherapy and assessment of toxicity/gene expression profiling.

χ^2^ = Chi-square test; C. I., confidence interval; tt, independent sample *t*-test.
